# Development of a modified 3D region proposal network for lung nodule detection in computed tomography scans: a secondary analysis of lung nodule datasets

**DOI:** 10.1186/s40644-024-00683-x

**Published:** 2024-03-20

**Authors:** Chia-Ying Lin, Shu-Mei Guo, Jenn-Jier James Lien, Tzung-Yi Tsai, Yi-Sheng Liu, Chao-Han Lai, I-Lin Hsu, Chao-Chun Chang, Yau-Lin Tseng

**Affiliations:** 1 Department of Medical Imaging, College of Medicine, National Cheng Kung University Hospital, National Cheng Kung University, No.1, University Road, 701 Tainan City, Taiwan; 2 Department of Computer Science and Information Engineering, National Cheng Kung University, Tainan, Taiwan; 3 Department of Surgery, College of Medicine, National Cheng Kung University Hospital, National Cheng Kung University, Tainan, Taiwan; 4 Division of Thoracic Surgery, Department of Surgery, College of Medicine, National Cheng Kung University Hospital, National Cheng Kung University, Tainan, Taiwan

**Keywords:** Anchor Assignment, Computer-aided detection system, Ground glass nodules, LUNA16 dataset, 3D region proposal network

## Abstract

**Background:**

Low-dose computed tomography (LDCT) has been shown useful in early lung cancer detection. This study aimed to develop a novel deep learning model for detecting pulmonary nodules on chest LDCT images.

**Methods:**

In this secondary analysis, three lung nodule datasets, including Lung Nodule Analysis 2016 (LUNA16), Lung Nodule Received Operation (LNOP), and Lung Nodule in Health Examination (LNHE), were used to train and test deep learning models. The 3D region proposal network (RPN) was modified via a series of pruning experiments for better predictive performance. The performance of each modified deep leaning model was evaluated based on sensitivity and competition performance metric (CPM). Furthermore, the performance of the modified 3D RPN trained on three datasets was evaluated by 10-fold cross validation. Temporal validation was conducted to assess the reliability of the modified 3D RPN for detecting lung nodules.

**Results:**

The results of pruning experiments indicated that the modified 3D RPN composed of the Cross Stage Partial Network (CSPNet) approach to Residual Network (ResNet) Xt (CSP-ResNeXt) module, feature pyramid network (FPN), nearest anchor method, and post-processing masking, had the optimal predictive performance with a CPM of 92.2%. The modified 3D RPN trained on the LUNA16 dataset had the highest CPM (90.1%), followed by the LNOP dataset (CPM: 74.1%) and the LNHE dataset (CPM: 70.2%). When the modified 3D RPN trained and tested on the same datasets, the sensitivities were 94.6%, 84.8%, and 79.7% for LUNA16, LNOP, and LNHE, respectively. The temporal validation analysis revealed that the modified 3D RPN tested on LNOP test set achieved a CPM of 71.6% and a sensitivity of 85.7%, and the modified 3D RPN tested on LNHE test set had a CPM of 71.7% and a sensitivity of 83.5%.

**Conclusion:**

A modified 3D RPN for detecting lung nodules on LDCT scans was designed and validated, which may serve as a computer-aided diagnosis system to facilitate lung nodule detection and lung cancer diagnosis.

**Supplementary Information:**

The online version contains supplementary material available at 10.1186/s40644-024-00683-x.

## Key message

The optimal 3D RPN for detecting lung nodules in chest CT was established.

The modified 3D RPN trained on the LUNA16 dataset had a higher CPM.

But CMP dropped if the modified 3D RPN trained on Taiwanese datasets.

**Supplementary Information:**

The online version contains supplementary material available at 10.1186/s40644-024-00683-x.

## Summary statement

A modified 3D RPN for detecting lung nodules on CT images that exhibited greater sensitivity and CPM than did several previously reported CAD detection models was established.

**Supplementary Information:**

The online version contains supplementary material available at 10.1186/s40644-024-00683-x.

## Introduction

Lung cancer is the most common cause of global cancer incidence and cancer-related deaths [1]. The aggressive and heterogeneous nature of lung cancer has precluded efforts to increase early detection via screening with chest radiography or sputum evaluation [2]. Clinical trials have proven that low-dose computed tomography (LDCT) for early lung cancer detection decreases mortality by 20% compared with chest X-rays [2]. LDCT provides detailed representation of the lung parenchyma and notable sensitivity to findings associated with early lung cancer, primarily lung nodules [3–5]. However, LDCT screening generates 300–500 images per patient, imposing an overwhelming burden on radiologists. To reduce this load, deep-learning techniques are being used to develop computer-aided detection (CAD) systems to screen for pulmonary nodules.

The main tasks of CAD systems for pulmonary nodule screening are nodule detection and characterization to eliminate false positives [6]. Deep-learning models for lung nodule analysis are trained to detect and classify nodules using a large set of labeled data (CT scans) and a convolutional neural network (CNN)-based algorithm [7, 8]. The performance of object detection systems has been improved by the addition of regional proposal networks (RPN) such as Faster R-CNN [9] that tell the CNN module where to look for objects.

Several lines of evidence indicated that 3D CNNs achieved higher competition performance metrics (CPMs) than their 2D counterparts for detecting lung nodules [10–13]. However, 3D CNNs are still in the initial stages of development [6, 11]. Several deep-learning techniques for 2D object detection, including the Residual Network (ResNet) module [14], the ResNeXt module [15], the Feature Pyramid Network (FPN) [5], and anchor assignment [16, 17], have been adapted successfully to improve the performance of 3D object detection. The development of 3D CAD systems for lung nodule detection was further promoted by the LUNA16 Challenge, which supplied the research community with a framework for testing and comparing algorithms on a common large database with a standardized evaluation protocol [18].

Using module substitution and pruning experiments, this study aims to develop a deep learning model for detecting pulmonary nodules in CT images with improved performance over existing systems by modifying the 3D RPN derived from a 2D object detection model based on Faster R-CNN, called RetinaNet [5, 9, 19]. By training and testing the model on 3 datasets representing patients with different demographic backgrounds, the study aims to broaden the application of the modified 3D RPN.

## Methods

### Lung nodule datasets

In this secondary data analysis, three datasets of lung modules acquired on LDCT were used to evaluate the performance of the modified 3D RPN in this study. The Lung Nodule Analysis 2016 (LUNA16) dataset is the largest public dataset, comprising 1186 lung nodules from 888 patients [18]. This dataset has been used widely to evaluate a variety of deep-learning–based pulmonary nodule detection methods [7, 20–22]. In addition, two private ongoing pulmonary nodule datasets maintained by the Radiology Department at the National Cheng Kung University Hospital (NCKUH) were used in this study: the NCKUH Lung Nodule received Operation (LNOP) dataset that included patients undergoing surgical resection for lung nodules with histological confirmation, and the NCKUH Lung Nodule in Health Examination (LNHE) dataset that included patients with lung nodules that were found by LDCT.

The LUNA16 dataset contains 1186 lung nodules. To minimize the bias caused by variation in nodule number, approximate 1000 pulmonary nodules were retrieved from LNOP and LNHE datasets. Therefore, the data of 1027 lung nodules derived from 708 patients, which were collected in the LNOP dataset from Dec. 2018 to Dec. 2021, were retrieved for training and testing deep learning models. In addition, the data of 1000 lung nodules derived from 420 patients, which were collected in the LNHE from Jan. 2019 to Dec. 2020, were used in this study.

Moreover, for temporal validation, the whole 1027 and 1000 lung nodules from LNOP and LNHE, respectively, were used as train sets. Additional 348 and 500 lung nodules that were recently collected in LNOP and LNHE, respectively, were used as test sets.

### Data annotation

The regions of interest (ROIs) of pulmonary nodules on axial images were manually labeled slice by slice by a thoracic radiologist (C.L.) and a thoracic surgeon (C.C.). After reaching consensus, 2D ROIs were converted to form 3D ROI. The 3D ROI of lung nodule was defined as the ground truth in this study.

### 3D region proposal network

**Fig. 1:**
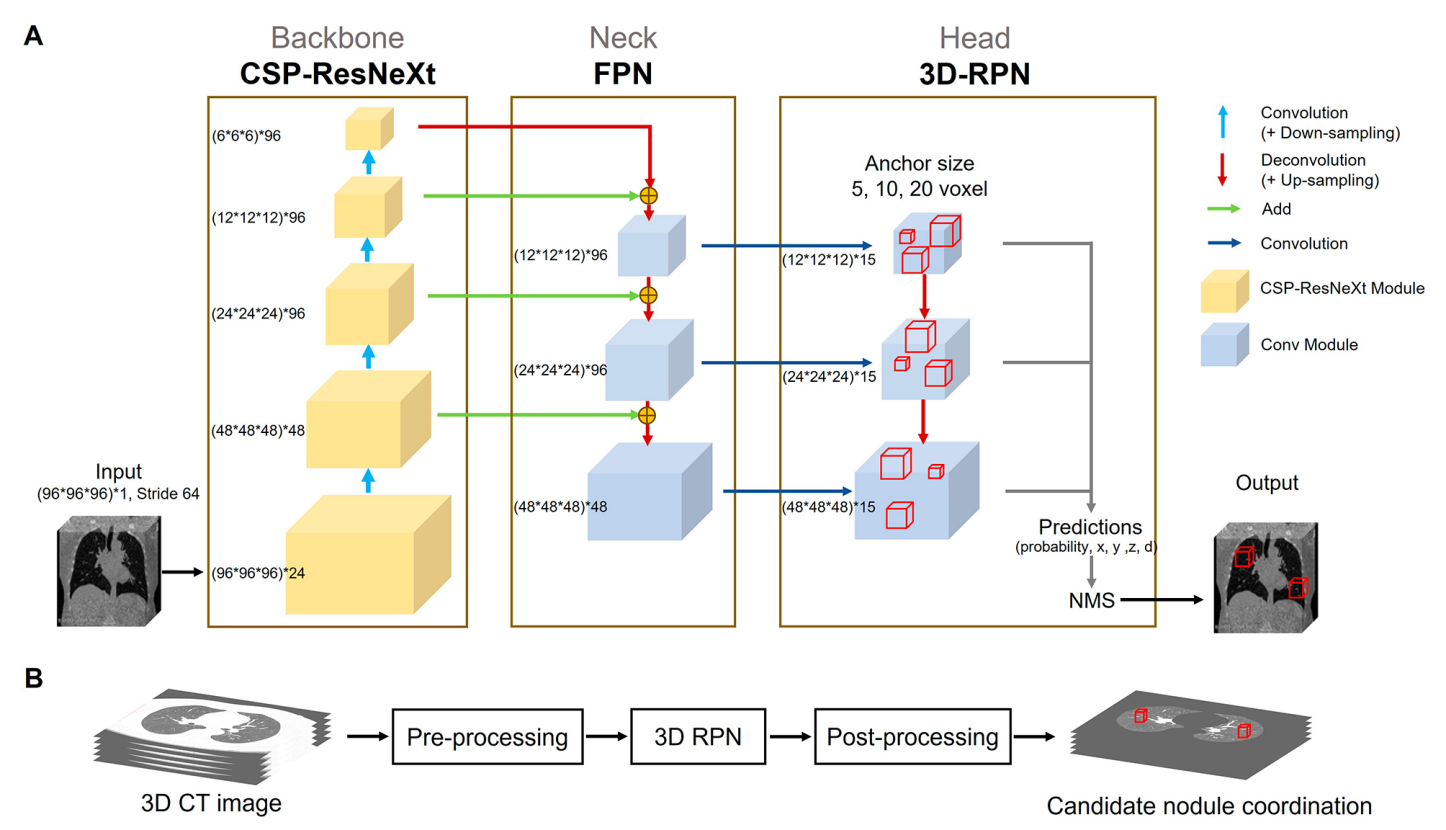
The architectural architecture of deep learning model. **(A)** 3D RPN. The boxes with anchor sizes of 5, 10, and 20 voxel sizes in each layer of detectors were used in the head block. Because the outputs included probability, x, y, z, d, the dimensions of each layer were 3* 5 = 15. **(B)** The complete pulmonary nodule detection system

The architecture of the proposed 3D RPN consisted of three blocks: backbone, neck, and head (Fig. 1A). The backbone network is used for feature extraction; the neck is used for feature fusion; and the head is used for dense prediction, which generates a prediction frame (anchor box) for each anchor point on the feature map. The training environment and training strategy is listed in Table 1.

**Table 1:** The training environment and training strategy

**Training environment**
OS: Windows 10 (64-bit)
CPU: Intel Core i7-9700 H @ 3.00 GHz
GPU: Single NVIDIA GeForce RTX 2070–8GB
RAM: 24GB
Deep learning framework: Pytorch
**Training strategy**
Optimizer: Ranger (with Gradient Centralization)
Learning rate: 0.001
Epoch: 200
Data augmentation method:
Randomly fliping in xy plane
Randomly rotating 0 ~ 180 degrees on the z-axis
Random scaling 0.75 ~ 1.25 times

### Architecture and modification of the pulmonary nodule detection system

The architecture of the 3D lung-nodule detection system is composed of three modules: pre-processing, deep learning model (3D RPN), and post-processing (Fig. 1B). 3D patch-based image input was adopted for pre-processing and post-processing. In the pre-processing module, to resample all CT images to the same size, the voxel spacing of all CT images was resampled to 1:1:1 mm. Each radiodensity value was converted from Hounsfield units (HU) (range, − 1200 to 600 HU) to a decimal between 0 and 1 and stored as a single-precision floating-point number. In the post-processing module, the extrapulmonary region is removed to reduce the false positive.

### Pruning experiments

**Fig. 2:**
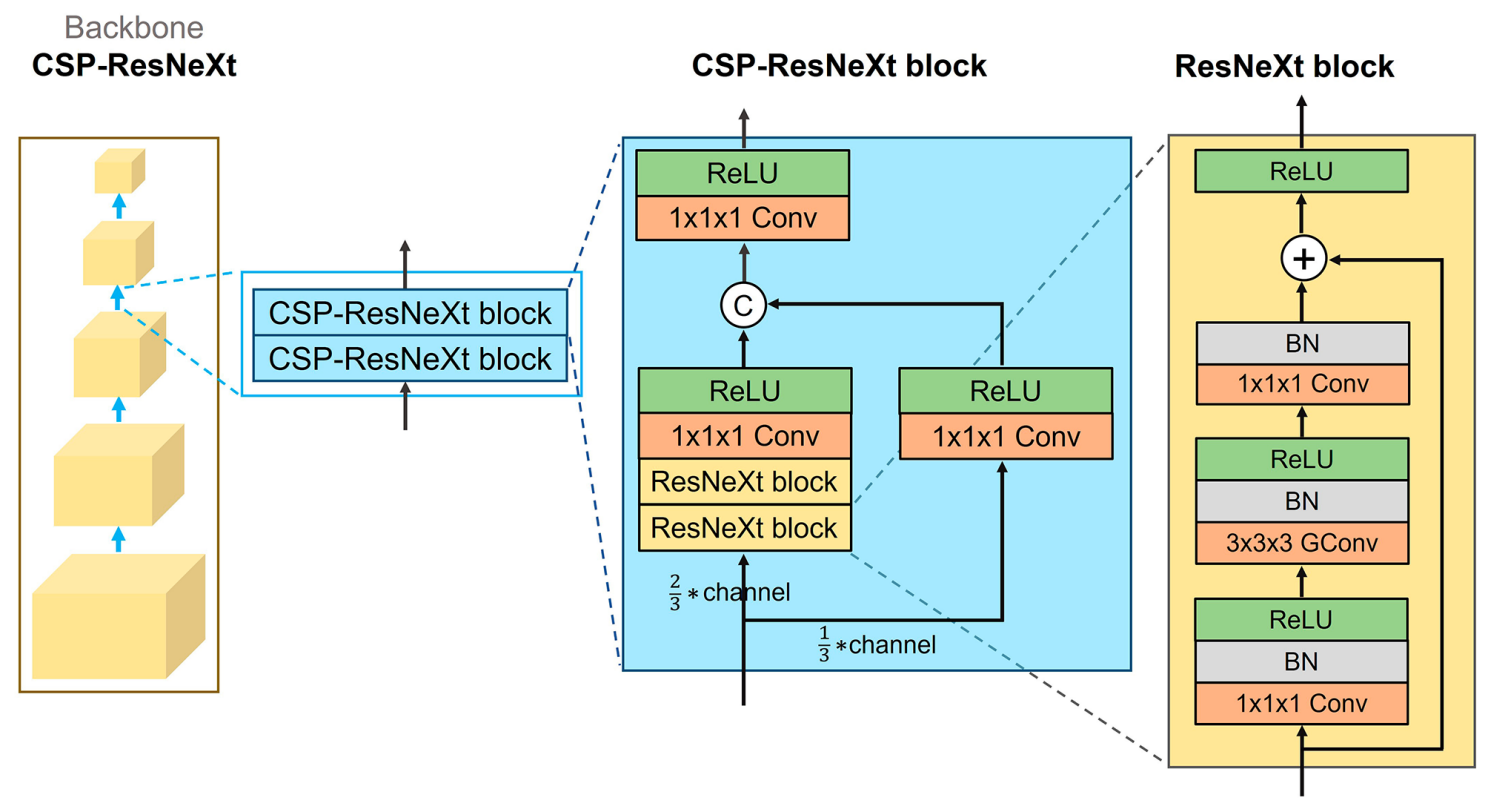
The CSPNet and ResNeXt modules are integrated into the design of the backbone network

In the training, a series of pruning experiments were performed using the LUNA16 dataset to modify each block of the 3D RPN for better performance. Although the ResNet module is commonly used to construct the backbone network [9], we first replaced the ResNet module with the ResNeXt module in the training phase [15]. Subsequently, the design of the Cross Stage Partial Network (CSPNet) [23], was incorporated into the ResNeXt module to form the CSP-ResNeXt module (Fig. 2). The FPN design was then added to the neck and detector of the selected 3D RPN with the CSP-ResNeXt module, achieving feature fusion and multi-level outputs on the neck and detector. The next pruning experiment involved modification of the anchor assignment of the 3D RPN.

### Nearest anchor assignment

**Fig. 3:**
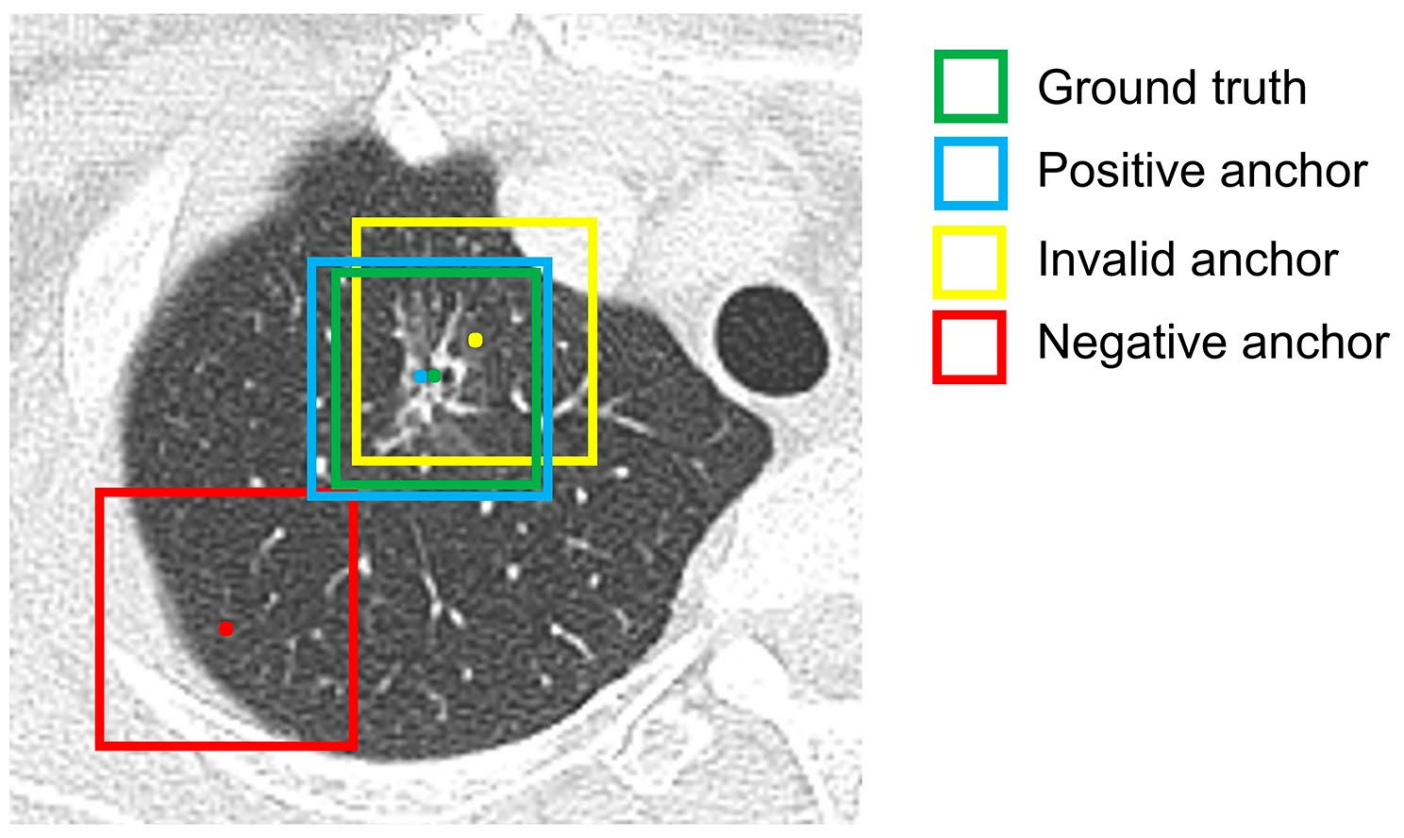
Illustration of the nearest anchor method. The IoU-based method could recognize both blue and yellow anchor boxes as positive samples. In contrast, the nearest anchor method recognized the blue anchor as the positive anchor, because it had an anchor point closest to the ground truth (green)

Anchor assignment, also called training sample selection, is the training of an object detection model to decide which anchor boxes on the input image patch are positive, negative, or ignored samples based on the ground truth in the training phase [9]. Only positive and negative samples are involved in the used for calculating the loss function. Because most lung nodules were almost spherical in shape with varied sizes, the boxes with anchor sizes of 5, 10, and 20 voxel sizes in each layer of detectors were used in the head block of 3D RPN (Fig. 1A). Several studies of object detection have used fixed Intersection over Union (IoU) matching for anchor assignment [5, 24]; however, the IoU matching method often results in multiple positive samples (Fig. 3).

To search for a more suitable anchor assignment method for 3D lung nodule detection, we applied the nearest anchor method in this study. The nearest anchor method assigned the only one anchor box with anchor point closest to the ground truth as the positive anchor (Fig. 3). If multiple anchor boxes shared a common anchor point, only the anchor box closest to the ground truth in size was selected as the positive sample.

### Performance evaluation measures

The modified 3D RPN was then trained on the LUNA16, LNOP, and LNHE datasets. The performance of the modified 3D RPN was evaluated by 10-fold cross-validation using free-response receiver operating characteristic (FROC) and CPM. The FROC is the curve drawn by the model showing the true positive rate under different confidence thresholds. The average recall rate (sensitivity) was defined at 0.125, 0.25, 0.5, 1, 2, 4, and 8 false positives per scan as previously described [25, 26]. CPM, a metric derived from FROC, was the average recall of 7 specific false positives per scan on the FROC. CPM and sensitivity were expressed as mean ± standard deviation (SD). After training, the modified 3D RPN was then tested on the LUNA16, LNOP, and LNHE test sets, with the average recall rate set at 2 false positives per scan.

## Results

### Comparison of pulmonary nodule characteristics between the three datasets

**Fig. 4:**
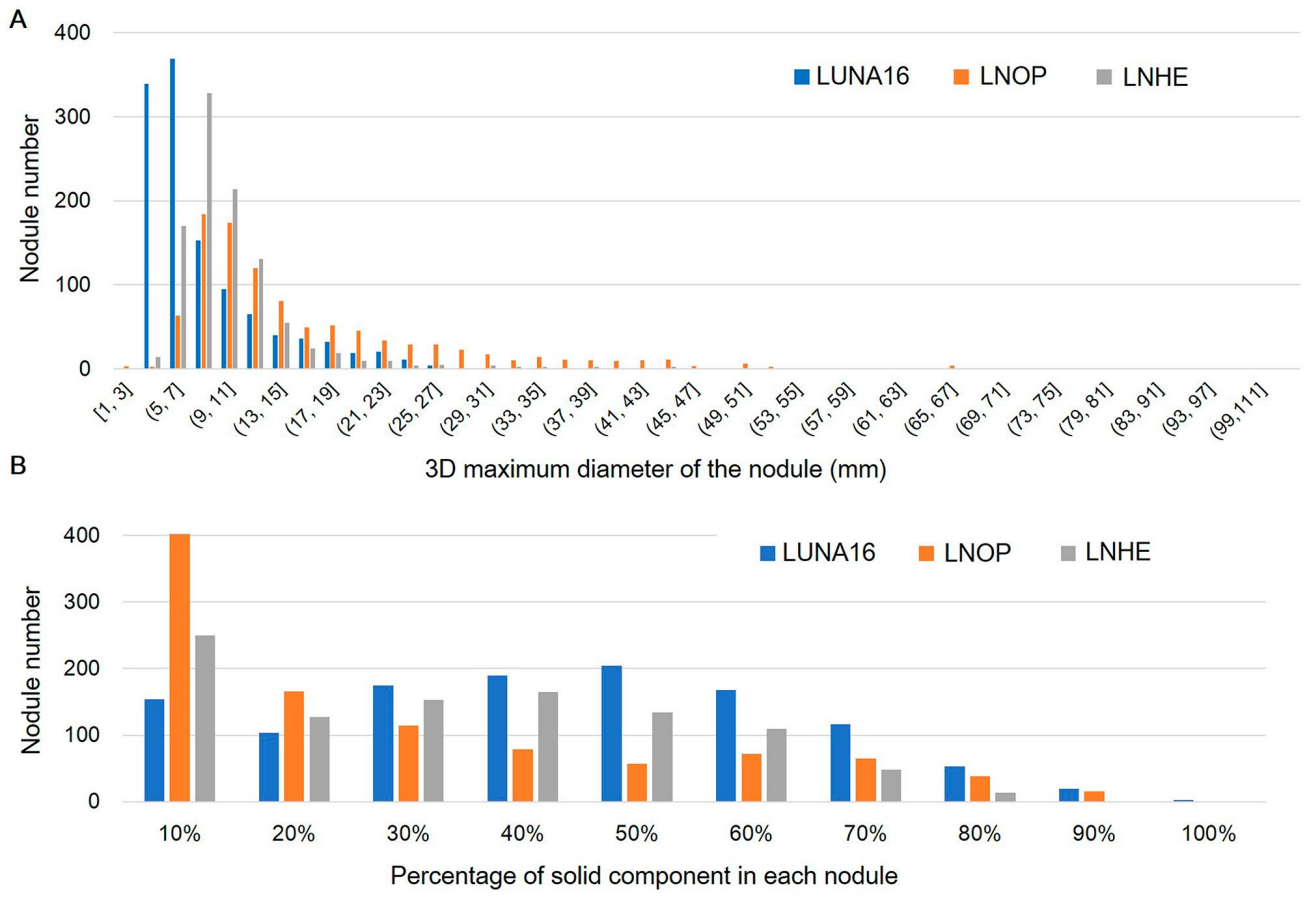
Characteristics of lung nodules in three datasets. **(A)** Distribution of 3D maximum diameter of each nodule. **(B)** Distribution of percentage of solid component in each nodule

The distribution of the 3D maximum diameter of each nodule for three datasets is shown in Fig. 4A. The LUNA16 dataset had a right-skewed distribution with a largest 3D maximum diameter of 32 mm. Lung nodules in the LNOP dataset were larger than those in the LUNA16 dataset, with the largest 3D maximum diameter at 93 mm. Lung nodules in the LNHE dataset were smaller than those in the LNOP dataset, with the largest 3D maximum diameter at 43 mm.

The distribution of solid-component percentage of each nodule in the LNOP dataset was right-skewed, with near 40% of nodules having 10% solid component (Fig. 4B). In contrast, the distribution of lung nodule solid-component percentage was relatively evenly distributed in the LUNA16 dataset (Fig. 4B).

### Modification of the 3D RPN

To improve the 3D RPN, a series of pruning experiments was conducted using the LUNA16 dataset. The performance evaluation revealed that the CPMs for the ResNet, ResNeXt, and CSP-ResNeXt modules were 86.8%, 88.2%, and 89.7%, respectively (Table 2). After adding the FPN design to the CSP-ResNeXt module, the CPM improved from 89.7 to 90.1% (Table 2). Although the IoU matching method has been widely used in several studies, the nearest anchor method achieved a slightly higher CPM (92.2% vs. 91.1%) (Table 2).

**Table 2:** Pruning experiments for modification of the 3D region proposal network

Backbone	FPN	Anchor Assignment	CPM
ResNet			86.8%
ResNeXt			88.2%
CSP-ResNeXt			89.7%
CSP-ResNeXt	✓		90.1%
CSP-ResNeXt	✓	IoU matching	91.1%
CSP-ResNeXt	✓	Nearest anchor	92.2%

Abbreviations: FPN, feature pyramid network; CMP, competition performance metric

### Performance of the modified 3D RPN

Of the three datasets, the modified 3D RPN trained on the LUNA16 dataset had highest sensitivities at various numbers of false positives per scan, while the modified 3D RPN trained on the LNHE dataset had the lowest sensitivities (Table 3). In addition, the modified 3D RPN trained on the LUNA16 dataset had the highest CPM (90.1%), followed by the LNOP (CPM, 74.1%) and the LNHE (CPM, 70.2%) (Table 3).

**Table 3:** Performance comparison of the modified 3D RPN trained on three datasets^**†**^

Number of false positives per scan	Sensitivity							CPM
	0.125	0.25	0.5	1	2	4	8	
Data source								
LUNA16	77.0%±2.9%	84.3%±2.6%	89.4%±2.4%	92.5%±1.9%	94.6%±1.5%	96.0%±1.0%	96.6%±0.7%	90.1%±1.4%
LNOP	45.9%±5.4%	58.3%±4.1%	67.4%±3.3%	76.9%±3.4%	84.8%±3.5%	91.0%±2.5%	94.6%±2.5%	74.1%±3.1%
LNHE	46.9%±3.4%	54.6%±5.5%	63.1%±4.3%	71.7%±3.0%	79.7%±3.2%	85.7%±2.2%	89.7%±2.9%	70.2%±2.1%

^**†**^Values represent the pulmonary nodule detection sensitivities (%) according to the number of false positives per CT scan

The results were expressed as mean ± SD.

Furthermore, the modified 3D RPN trained and tested on the same datasets had sensitivities of 94.6%, 84.8%, and 79.7% for LUNA16, LNOP, and LNHE, respectively (Table 4). The sensitivity dropped substantially if the test set differed from the training set.

**Table 4:** Sensitivity comparison of the modified 3D PRN trained and tested on various combinations of datasets at 2 false positives per scan

		Training dataset		
		LUNA16	LNOP	LNHE
Test dataset	LUNA16	94.6% ± 1.5%	63.4% ± 2.6%	45.5% ± 1.7%
	LNOP	78.6% ± 2.3%	84.8% ± 3.5%	50.0% ± 3.7%
	LNHE	67.0% ± 2.2%	78.2% ± 2.5%	79.7% ± 3.2%

The results were expressed as mean ± SD.

### Temporal validation

To confirm the predictive performance of the modified 3D RPN, temporal validation was performed. The modified 3D RPN tested on LNOP and LNHE test sets achieved CPM of 71.6% and 71.7% (Table 5). The CMP of modified 3D RPN on LNOP test set slightly decreased from 74.1 to 71.6%, while the CMP of modified 3D RPN on LNHE test set slightly increased from 70.2 to 71.7%. Under the most clinically acceptable condition (false positive per scan = 2), the sensitivity of LNOP test set increased from 84.8 to 85.7%, and the sensitivity of LNHE test set increased from 79.7 to 83.5% (Table 5).

**Table 5:** Performance of the modified 3D RPN on test datasets^**†**^

Number of false positives per scan	Sensitivity							CPM
	0.125	0.25	0.5	1	2	4	8	
Test dataset								
LNOP	44.5%	53.7%	59.2%	76.9%	85.7%	91.6%	94.1%	71.6%
LNHE	45.3%	53.2%	62.6%	74.8%	83.5%	88.5%	94.2%	71.7%

^**†**^Values represent the pulmonary nodule detection sensitivities (%) according to the number of false positives per CT scan

### The influence of solid components of nodules

To assess the extent to which percentages of solid components of lung nodules affect the predictive performance of the modified 3D RPN, stratification analyses were performed. Each of the three datasets, LUNA16, LNOP, and LNHE, was stratified by the percentages of solid components of nodules into three ranges: 0 to 10%, 10 to 50%, and 50 to 100%. Subsequently, the performance of the modified 3D RPN trained on each dataset stratified by the solid component range was examined. Within each data source, the performance of the modified 3D RPN increased with the percentages of solid components of nodules (Table 6). Among data sources, LUNA16 had higher CPM rates than LNOP and LNHE.

**Table 6:** Performance comparison of the modified 3D RPN trained on three datasets stratified by the range of solid components of nodules

Number of false positive per scan		Sensitivity							CPM
		0.125	0.25	0.5	1	2	4	8	
Data source	Solid component range								
LUNA16	0 to 10%	72.1%	0.779	81.7%	84.6%	85.6%	88.5%	88.5%	82.7%
	10 to 50%	81.3%	0.859	93.0%	94.5%	96.1%	96.1%	96.1%	91.9%
	50 to 100%	80.4%	0.893	92.9%	92.9%	95.5%	97.3%	97.3%	92.2%
LNOP	0 to 10%	35.0%	41.7%	53.3%	66.7%	83.3%	91.7%	95.0%	66.7%
	10 to 50%	45.8%	56.2%	68.8%	78.4%	88.1%	95.9%	97.9%	75.9%
	50 to 100%	45.3%	60.6%	67.2%	80.4%	91.2%	96.4%	98.9%	77.1%
LNHE	0 to 10%	42.9%	45.7%	60.0%	74.3%	77.1%	85.7%	91.4%	68.2%
	10 to 50%	53.8%	55.8%	61.5%	75.0%	78.8%	88.5%	94.2%	72.5%
	50 to 100%	51.6%	57.9%	63.6%	79.3%	88.3%	90.9%	94.5%	75.2%

## Discussion

Pruning experiments on the 3D RPN with module substitutions showed that the optimal 3D RPN contained the CSP-ResNeXt module, FPN, nearest anchor method, and post-processing masking, achieving a CPM of 91.1%. The modified 3D RPN trained on the LUNA16 dataset had the highest CPM (90.1%), followed by the LNOP (74.1%) and the LNHE (70.2%) datasets. The modified 3D RPN trained and tested on the same dataset had the highest sensitivity (LUNA16, 94.6%; LNOP, 84.8%; LNHE, 79.7%). Furthermore, the reliability of the modified 3D RPN was confirmed by temporal validation.

Comparison of pulmonary nodule characteristics between the three datasets showed that nodules in the CT images from patients in the LNOP dataset (Taiwanese patients with histologically-confirmed lung nodules) and LNHE dataset (Taiwanese patients with nodules found during health examination) were larger and had a greater non-solid component than did those in the LUNA16 dataset (Western patients) (Fig. 4). This finding is consistent with reports showing that Asian patients tend to have a higher proportion of non-solid pulmonary nodules [27], which have a ground-glass opacity on CT images [28]. This difference in nodule properties between populations may contribute to the differences in performance of our model on the 3 datasets, suggesting the importance of considering ethnicity factors in datasets used for training and testing diagnostic deep-learning models.

Accurate classification of ground glass nodules is of great therapeutic value, as they are associated with both benign inflammatory conditions and various types of malignancy [29, 30]. In addition, consolidation-to-tumor ratio is positively associated with tumor invasiveness [31]. Because of their optical properties, ground glass nodules may be undetected in CT scans, and deep-learning algorithms are being developed to distinguish these nodules from surrounding tissues [23, 32]. Our finding that the LNOP dataset is enriched in data for non-solid nodules as compared to the LUNA16 dataset suggests that the LNOP may be useful in the development of algorithms for detecting and classifying ground glass nodules.

As shown in Supplementary Table S1, the CPM and sensitivity of our modified Faster R-CNN-based 3D RPN on the LUNA16 dataset surpassed that of other CAD models for lung nodule detection. DeepLung [22], a 3D Faster R-CNN designed for nodule detection with 3D dual path blocks and a U-net-like encoder-decoder structure and a gradient boosting machine with 3D dual path network features for nodule classification; DeepSEED [20], which has an encoder-decoder structure in conjunction with a RPN and uses dynamically-scaled cross entropy loss to reduce false positives and combat the sample imbalance problem associated with nodule detection; CPM-Net [7], a 3D center-points matching detection network that is anchor-free and automatically predicts the position, size, and aspect ratio of nodules; and SCPM-Net [21], a 3D sphere representation-based center-points matching detection network that is anchor-free and automatically predicts the position, radius, and offset of nodules without manual design of nodule/anchor parameters.

The present secondary data analysis has several limitations. The modified 3D RPN model is complex, containing 1,284,508 parameters, requiring about 80 hours to perform 10-fold cross validation on a dataset of 1000 lung nodules. In future studies, we aim to shorten the training time by simplifying the model without sacrificing specificity. In addition, it has been reported that CT manufacture did not affect performance of deep leaning model for detecting lung nodules [33]. In contrast, reconstruction kernel affected texture features and wavelet features of CT images [34], and the poor image quality resulted in more false positives per scan. The investigation of the influence of CT hardware, reconstruction kernels, and image quality on performance of the modified 3D RPN will be another future research direction. Furthermore, the improved 3D RPN model will be trained on the updated LNOP and LNHE datasets with more lung nodule data. We will also try to access more powerful hardware to speed up the lung nodule detection process. To reduce false positives, we will add a false-positive reduction model to the modified 3D RPN model.

## Conclusion

The modified 3D RPN model trained on the LUNA16 dataset exhibited a sensitivity of 96.6% at 8 false positives per scan and a CPM of 90.1%, which may serve as a potential CAD tool to facilitate lung nodule detection and of lung cancer diagnosis. In addition, the difference in performance between datasets comprising Western and Asian patients indicates the need for establishing training and testing datasets specific to Asian patients. The LNOP dataset may be useful for training and testing CAD models to identify lung nodules with ground glass opacity, which are associated with malignancy and tumor invasiveness.

### Electronic supplementary material

Below is the link to the electronic supplementary material.

Supplementary Material 1

## Data Availability

The LNOP and LNHE datasets are not publicly available; the data are available from the corresponding author (C.C), upon reasonable request.

## Abbreviations

CAD: computer-aided detection
CNN: convolutional neural network
CPM: competition performance metric
CSPNet: Cross Stage Partial Network
CSP-ResNeXt: CSPNet approach to ResNeXt
DL: deep learning
FPN: feature pyramid network
FROC: free-response receiver operating characteristic
HU: Hounsfield unit
IoU: intersection over Union
IRB: Institutional Review Board
LDCT: low-dose computed tomography
LNHE: Lung Nodule in Health Examination
LNOP: Lung Nodule Received Operation
LUNA16: Lung Nodule Analysis 2016
NCKUH: National Cheng Kung University Hospital
ResNet: Residual Network
ROI: region of interest RPN:region proposal network
SD: standard deviation
