# Supplementary material for: Development of a modified 3D region proposal network for lung nodule detection in computed tomography scans: a secondary analysis of lung nodule datasets
Source: Cancer Imaging. 2024 Mar 20;24:40. doi: 10.1186/s40644-024-00683-x (PMC10953193; doi:10.1186/s40644-024-00683-x)
Supplement: Supplementary file 1 — Supplementary Material 1 [file 40644_2024_683_MOESM1_ESM.docx]

**Supplementary Table S1.** Performance comparison between the modified 3D PRN and previously-reported lung-nodule–detection models on the LUNA16 dataset.*

| Number of false positives per scan | **Sensitivity** | | | | | | | **CPM** |
| --- | --- | --- | --- | --- | --- | --- | --- | --- |
|  | 0.125 | 0.25 | 0.5 | 1 | 2 | 4 | 8 |  |
| **Deep learning models** |  |  |  |  |  |  |  |  |
| DeepLung (2018) [21] | 69.2% | 76.9% | 82.4% | 86.5% | 89.3% | 91.7% | 93.3% | 84.2% |
| DeepSEED (2020) [19] | 73.9% | 80.3% | 85.8% | 88.8% | 90.7% | 91.6% | 92.0% | 86.2% |
| CPM-Net (2020) [7] | 72.3% | 83.8% | 88.7% | 91.1% | 92.8% | 93.4% | 94.8% | 88.1% |
| SCPM-Net (2022) [20] | 74.3% | 82.9% | 88.9% | 92.2% | 93.9% | 95.8% | 96.4% | 89.2% |
| Modified 3D RPN | 77.0% | 84.3% | 89.4% | 92.5% | 94.6% | 96.0% | 96.6% | 90.1% |

*****The pulmonary nodule detection sensitivity (%) was determined according to the number of false positives per CT scan.
